# Color-associated metabolic divergence and nutritional variation in colored cauliflower curds

**DOI:** 10.1016/j.fochx.2026.104197

**Published:** 2026-07-09

**Authors:** Difei Wang, Meijiao Lu, Zitong Zhao, Laibin Cheng, Wanqing Dong, Jin Cui, Zhenguo Shen, Nana Su

**Affiliations:** a College of Life Sciences, Nanjing Agricultural University, Nanjing 210095, China; b Shanxi Huazhichuang Agricultural Science and Technology Co., Ltd, Baoji 721306, China; c National Demonstration Center for Experimental Biology Education, Zhejiang University, Hangzhou 310058, China

**Keywords:** Cauliflower (*Brassica oleracea* var. *botrytis*), Curd color, Non-targeted metabolomics, Nutritional quality, Functional food, Phytochemicals

## Abstract

Cauliflower curd color influences nutritional quality and consumer preference. Using non-targeted metabolomics, we profiled four cultivars (white, green, yellow, gold). White curds accumulated more soluble sugars, while colored curds showed elevated tricarboxylic acid (TCA) cycle intermediates and secondary metabolites. Green curds were enriched for chlorophyll-associated amino acids, sulforaphane and gamma-aminobutyric acid (GABA), whereas yellow/gold curds accumulated more flavonoids and phenolic acids. These metabolic differences were linked to enhanced antioxidant potential and flavor diversity. This hypothesis-generating study provides a metabolic basis for breeding nutritionally enhanced cauliflower varieties.

## Highlights

• First comparative metabolomics of white, green, yellow, and gold cauliflower.
• White curds accumulate soluble sugars (energy-storage mode).
• Colored curds shift to TCA cycle and amino acid biosynthesis.
• Green curds enrich sulforaphane and GABA.
• Yellow/gold curds enrich flavonoids and phenolic acids.

## Graphical abstract

**Figure:**
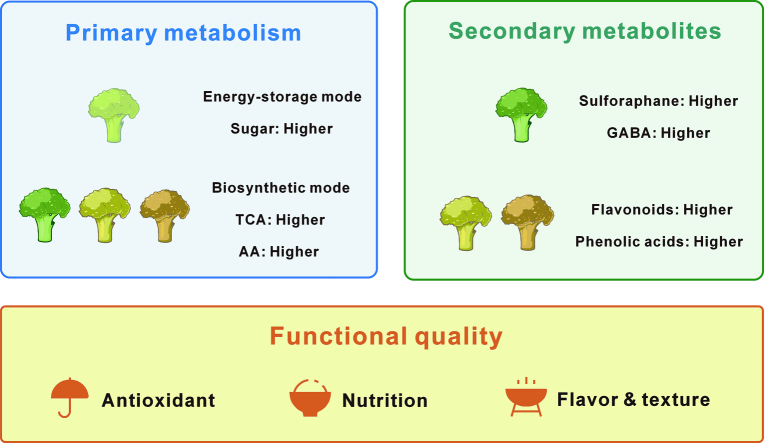

## 1 Introduction

Cauliflower is a nutrient-dense vegetable rich in proteins, vitamins, minerals, dietary fiber, and bioactive substances (such as glucosinolates, phenolic compounds and flavonoids), which have important health-promoting effects, including antioxidant, anti-inflammatory, and anti-cancer activities (Collado-González, Piñero, Otálora, López-Marín, & del Amor, 2021; Drabińska, Jeż, & Nogueira, 2021; Manchali, Chidambara Murthy, & Patil, 2012; Picchi et al., 2012; Sheng et al., 2025). The curd color of cauliflower is a key visual trait that affects consumer preference and market value, and different color cultivars also show differences in nutritional composition and functional characteristics (Chen et al., 2022; Chiu et al., 2010; Li, Paolillo, Parthasarathy, DiMuzio, & Garvin, 2001; Sheng et al., 2025). Currently, white cauliflower is the predominant commercial cultivar worldwide. Green cauliflower is a well-established but less common commercial specialty. In comparison, yellow and gold cauliflower are rare and are valued for their unique nutritional profiles associated with specific pigment types (betaxanthins and betacyanins), which are characteristic of yellow and gold coloration in various plant species (Koss-Mikołajczyk, Kusznierewicz, Wiczkowski, Płatosz, & Bartoszek, 2019; Sheng et al., 2025; Shrawan Singh, Kalia, & Selvakumar, 2025).

The color formation of cauliflower curds is closely related to the synthesis and accumulation of pigments, among which chlorophyll is the main pigment determining green color, while betaxanthin and betacyanin are important pigments involved in the formation of yellow and gold colors in plants. In cauliflower, these color phenotypes are associated with differential accumulation of aromatic amino acid precursors for these pigment pathways, as explored in this study (Shubham Singh, Yadav, & Singh, 2021; Tanaka, Sasaki, & Ohmiya, 2008). The relationship between free amino acid composition and curd color in cauliflower remains poorly understood. (Park et al., 2013; Roy, Basak, Mainak, & Islam, 2025; Sheng et al., 2025). Beyond its application in nutritional quality assessment, metabolomics has also emerged as a valuable tool for evaluating the impact of environmental factors—including emerging contaminants—on the biochemical composition of food crops. Recent studies have demonstrated that inorganic contaminants, such as nanomaterials, can accumulate through food webs and induce metabolic disturbances in exposed organisms (Hashemi et al., 2024). These findings highlight the importance of comprehensive metabolomic characterization of food resources, as metabolite composition may influence both nutritional quality and biological resilience under changing environmental conditions. Therefore, understanding the intrinsic metabolic diversity of crops like cauliflower also provides a baseline for assessing their responses to environmental stressors.

Non-targeted metabolomics is a powerful tool for analyzing the overall metabolite profile of organisms, which can systematically identify differential metabolites among different samples and explore the metabolic pathways involved in specific traits (Gorrochategui, Jaumot, Lacorte, & Tauler, 2016; Souza & Patti, 2021). Unlike targeted approaches, untargeted metabolomics captures a broad spectrum of metabolites without prior selection, enabling the discovery of unexpected metabolic changes associated with phenotypic variation. Recent studies have demonstrated the utility of this approach in characterizing metabolic networks underlying nutritional quality and bioactive compound accumulation in various food crops (Flores et al., 2025; Nepal, Rai, & Rai, 2025; Ra et al., 2025). When combined with multivariate statistical analysis and pathway enrichment, untargeted metabolomics allows for the systematic elucidation of metabolic networks linked to trait differentiation, such as color formation and nutritional quality. In recent years, this technology has been widely used in the study of vegetable quality, color formation, and stress response, but few studies have systematically analyzed metabolite differences and nutritional correlations among cauliflower cultivars with different curd colors (Koss-Mikołajczyk et al., 2019; Park et al., 2013; Sheng et al., 2025).

In this study, non-targeted metabolomic analysis was carried out on four cauliflower cultivars with different curd colors (white, green, yellow, and gold). The specific objectives were: (1) To identify differential metabolites among different color cauliflower curds, especially amino acids that serve as known precursors for pigment biosynthesis; (2) To explore precursor-based relationships with pigment phenotypes, and clarify their potential metabolic association with curd color formation, without establishing causality; (3) To analyze the changes of nutritional and flavor-related metabolites with the deepening of curd color, and explore how metabolite changes may relate to taste, flavor, and nutritional value of cauliflower based on metabolomic data. This study provides a systematic characterization of the phytochemical profiles associated with different curd colors in cauliflower. From a food chemistry perspective, these findings offer a scientific basis for selecting cauliflower varieties with specific nutritional attributes, such as high antioxidant capacity (gold curds) or high sulforaphane content (green curds), and support the development of color-specific functional cauliflower products (Park et al., 2013; Sheng et al., 2025; H. Yu et al., 2026). This study aims to systematically characterize the phytochemical profiles associated with different curd colors (white, green, yellow, and gold) in cauliflower using untargeted metabolomics, and to provide a metabolic basis for breeding nutritionally enhanced and color-specific functional cauliflower varieties.

## 2 Materials and methods

### 2.1 Plant materials

Four cauliflower cultivars with different curd colors were used in this study: white curd (H1041), green curd (H1031), yellow curd (H1009), and gold curd (H1003). All cultivars were planted in the experimental field of Shanxi Huazhichuang Agricultural Science and Technology co., Ltd. under the same cultivation conditions (uniform soil, irrigation, fertilization, and pest control) to avoid the influence of environmental factors on curd metabolism. When the curds reached commercial maturity (7–8 days after curd formation), three biological replicates were collected for each cultivar, with 3 curds per replicate. The curd samples were quickly frozen in liquid nitrogen after harvesting, and stored at −80 °C for subsequent metabolomic analysis.

### 2.2 Sample preparation and metabolites extraction for metabolomic analysis

Approximately 1 g of fresh tissue was excised from the curd, rinsed immediately with physiological saline to remove surface impurities, and blotted dry. The prepared tissue was wrapped in aluminum foil labeled with the corresponding sample name, flash-frozen in liquid nitrogen for 3–4 h, transferred to dry ice, and shipped to LC-Bio Technologies Co., Ltd. (Le Boulch et al., 2026; Yang et al., 2018; Q. Zhang et al., 2022). Weigh 50 mg (±5 mg) of the sample and add 500 μL of 80% icy methanol solution. Put a small amount of steel balls and grind them with a grinder, and incubated for 30 min at −20 °C to precipitate proteins, and centrifuged at 20000*g* for 10 min at 4 °C. The supernatant was centrifuged for 5 min again. The supernatant was transferred to a fresh vial for UPLC-HRMS analysis (Guerrieri et al., 2021; Y & Seelam, 2025). Methanol-based extraction was employed for its efficiency in recovering a broad range of polar and semi-polar metabolites, as recommended in recent metabolomics studies (Kadir, Malaka, Nahariah, Wahniyathi, & Arief, 2025; Rueangsri, Lasunon, Kwantrairat, & Taweejun, 2025). The quality control (QC) sample was prepared by mixing an equal aliquot of the supernatant of samples (Begou, Gika, Theodoridis, & Wilson, 2025).

### 2.3 Chromatography-mass spectrometry conditions

Metabolite separation was performed using a Thermo Vanquish Flex UPLC system equipped with an ACQUITY UPLC HSS T3 column (100 mm × 2.1 mm, 1.8 μm, Waters). The mobile phase comprised 5 mmol/L ammonium acetate and 5 mmol/L acetic acid in water (phase A) and acetonitrile (phase B), with gradient elution as follows: 0–0.8 min (2%–70% B), 0.8–2.8 min (70%–90% B), 2.8–5.3 min (90%–99% B), 5.3–5.9 min (99% B), 5.9–7.5 min (99%–2% B), 7.5–7.6 min (2% B), and 7.6–10.0 min (2% B). The flow rate was 0.35 mL/min, injection volume was 4 μL, and column temperature was maintained at 40 °C. A Thermo Q-Exactive Plus high-resolution tandem mass spectrometer was used for metabolite detection in both positive and negative electrospray ionization modes. The ESI temperature was 350 °C, with spray voltages of +3800 V (positive) and −3400 V (negative); sweep gas, auxiliary gas, and sheath gas pressures were set to 0 Arb, 15 Arb, and 50 Arb, respectively. Mass spectra were acquired in full scan and data-dependent acquisition (DDA) modes: full scan covered 70–1050 Da at 70 k resolution, AGC target 3E6, maximum IT 100 ms; the top 5 intense ions (>100,000 counts) were subjected to DDA scanning at 17.5 k resolution, maximum IT 50 ms, and dynamic exclusion 6 s.

### 2.4 Data processing and analysis

The acquired MS data pretreatments including peak picking, peak grouping, retention time correction, second peak grouping, and annotation of isotopes and adducts was performed using XCMS software (Karaman, Climaco Pinto, & Graça, 2018; Wen, Mei, Zeng, & Liu, 2017; Huaxu Yu et al., 2025). LC − MS raw data files were converted into mzXML format and then processed by the XCMS, CAMERA and metaX toolbox implemented with the R software(Wen et al., 2017). Each ion was identified by combining retention time (RT) and *m*/*z* data. Intensities of each peak were recorded and a three-dimensional matrix containing arbitrarily assigned peak indices (retention time-m/z pairs), sample names (observations) and ion intensity information (variables) was generated (Hoffmann et al., 2012; Huaxu Yu et al., 2025).

The online Kyoto Encyclopedia of Genes and Genomes (KEGG) and Human Metabolome Database (HMDB) were used to annotate the metabolites by matching the exact molecular mass data (m/z) of samples with those from database. If a mass difference between observed and the database value was less than 10 ppm, the metabolite would be annotated and the molecular formula of metabolites would further be identified and validated by the isotopic distribution measurements. We also used an in-house fragment spectrum library of metabolites to validate the metabolite identification (Cai, Zhou, & Zhu, 2023; Xiao, Zhou, & Ressom, 2012).

Statistical analysis was primarily conducted using R (v4.0). Metabolite data underwent three key processing steps: first, data filtering to remove samples with over 80% missing values or quality control (QC) samples with over 50% missing data; second, data imputation using the K-nearest neighbor (KNN) method; and third, data standardization via Probabilistic quotient normalization (PQN). Untargeted metabolomics data processing and statistical analysis were performed following established workflows for plant metabolomic (Fatima, Waheed, & Hassan, 2024; Nepal et al., 2025). Cluster heatmaps were generated with the R package pheatmap (Pang et al., 2024; Sun & Xia, 2024). Principal component analysis (PCA) and significant differential metabolite analysis were performed using the R package metaX. Partial least squares discriminant analysis (PLS-DA) was carried out with the R package ropls, and variable importance in projection (VIP) values for each variable were calculated. Correlation analysis was conducted using Pearson's correlation coefficient from the R package cor. The final significant differential metabolites were identified based on three criteria: *P*-value <0.05 from *t*-test, fold change >1.2 or < 0.833, and VIP ≥ 1 from PLS-DA analysis(Mock et al., 2018; Sun & Xia, 2024; Xu et al., 2024). KEGG pathway enrichment analysis was performed using hypergeometric tests, with P-value<0.05 indicating significant enrichment. Metabolite set enrichment analysis was conducted using GSEA (v4.1.0), and KEGG pathways with |NES| >1, a nominal P-value <0.05 and FDR < 0.25 were considered significantly different between the two groups. Network diagrams were constructed based on the pathways of the metabolites to illustrate their interactions (Cai et al., 2023; Muley, 2025; Ogata et al., 1999; L. Zhang et al., 2025).

## 3 Results

### 3.1 Metabolite profiling of different color cauliflower curds

This study analyzed four varieties of cauliflower (*Brassica oleracea* var. *botrytis*) exhibiting distinct curd colors: white (H1041), green (H1031), yellow (H1009), and gold (H1003). A total of 2685 metabolites were identified in the curds of four cauliflower cultivars by non-targeted metabolomic analysis, including 771 Lipids and lipid-like molecules, 701 Organoheterocyclic compounds, 464 Organic acids and derivatives, 227 Benzenoids, 186 Organic oxygen compounds, 125 Phenylpropanoids and polyketides, 69 Organic nitrogen compounds, 57 Alkaloids and derivatives, 26 Nucleosides, nucleotides, and analogues, 16 Organosulfur compounds. KEGG enrichment analysis revealed that metabolites were mainly enriched in Metabolic pathways, Biosynthesis of secondary metabolites, Biosynthesis of cofactors, Biosynthesis of amino acids, and Carbon metabolism (Fig. S1).

PLS-DA was applied to visualize the metabolic profiles, with PC1 and PC2 explaining 26.12% and 14.93% of the total variation, respectively (Fig. S2A). Biological replicates of each cultivar formed tight clusters, while samples of different color cultivars were clearly separated, indicating significant metabolic divergence among groups (Sheng et al., 2025). QC samples were closely clustered, verifying the stability of the analytical platform. A permutation test was further performed to validate the PLS-DA model (Fig. S2B). The original model exhibited higher R^2^ and Q^2^ values than all permuted models, with a Q^2^ intercept of −1.2394, confirming the absence of overfitting. These results demonstrated that the PLS-DA model was robust and reliable, enabling the subsequent identification of differential metabolites associated with curd color and nutritional quality.

Building on this, differential expression analysis was conducted to compare the metabolic profiles of each colored cultivar with the white control (H1041). As quantified in Fig. S3A, gold curds (H1003) exhibited 437 up-regulated and 592 down-regulated differentially accumulated metabolites (DAMs); yellow curds (H1009) showed 415 up-regulated and 437 down-regulated DAMs; and green curds (H1031) displayed 484 up-regulated and 574 down-regulated DAMs. The Venn diagram (Fig. S3B) further illustrated the overlap of DAMs among the three colored groups: a core set of 542 DAMs (39.71%) were commonly regulated across all three comparisons, while 146, 122, and 65 DAMs were uniquely altered in gold, green, and yellow curds, respectively.

To further identify significant DAMs and their related biological pathways, differential analysis and KEGG pathway enrichment were performed (Fig. S4). Volcano plots (Fig. S4A, C, E) clearly visualized the number and distribution of significantly up- and down-regulated metabolites in each colored curd. KEGG enrichment analysis (Fig. S4B, D, F) showed that DAMs in green curds were mainly enriched in metabolic pathways, biosynthesis of secondary metabolites, and amino acid metabolism, which are closely related to chlorophyll biosynthesis. In contrast, DAMs in yellow and gold curds were significantly enriched in aromatic amino acid biosynthesis associated with betalain biosynthesis pathways (Lopez-Nieves et al., 2018; Roca & Pérez-Gálvez, 2021; Tossi, Martínez Tosar, Pitta-Álvarez, & Causin, 2021).

To further reveal the metabolic differences among the three colored cauliflower curds, pairwise comparisons were performed among H1003, H1009, and H1031 (Fig. S5). Volcano plots (Fig. S5A, C, E) clearly showed the distribution of significantly up- and down-regulated metabolites in each pairwise comparison. KEGG enrichment analysis (Fig. S5B, D, F) revealed that DAMs between gold/green and yellow/green curds were mainly enriched in amino acid metabolites related to chlorophyll metabolism, and phenylpropanoid biosynthesis (Peng et al., 2025; Wang et al., 2020). DAMs between gold and yellow curds were primarily enriched in phenylpropanoid biosynthesis and aromatic amino acid biosynthesis, which provide important precursors for pigment accumulation (Peng et al., 2025). These results further confirm the distinct metabolic characteristics among different colored curds and provide a comprehensive metabolic basis for the formation of different curd colors.

These global metabolic differences provide a basis for further investigation of pigment-associated amino acid metabolism and nutritional characteristics in cauliflower curds of different colors (Rammohan, Reddy, Padmaja, Prasad, & Reddy, 2025; Z.-H. Zhang et al., 2024).

### 3.2 Differenial amino acids related to pigment synthesis

Although direct detection of chlorophylls and betalains was not achieved—a recognized limitation of untargeted LC-MS metabolomics due to their poor ionization efficiency—we identified 32 free amino acids, including well-established pigment precursors. Amino acids are key upstream metabolites involved in the biosynthesis of plant pigments, and their differential accumulation is closely associated with the variation of curd color in cauliflower (Ahmad et al., 2025). To clarify that are visually associated with curd color, it is critical to first identify and quantify free amino acids, followed by differential and correlation analyses. The specific research procedures are detailed in the following sections.

In this study, a total of 32 free amino acids were identified and quantified based on MS/MS spectrum matching against database records, including both proteinogenic and non-proteinogenic amino acids such as GABA. These amino acids were subsequently used in correlation analyses with pigment accumulation (Shen et al., 2025; Xue et al., 2025). Differentially accumulated amino acids were screened with the values of FC ≥ 1.2 or FC ≤ 0.833, *p* < 0.05, VIP ≥ 1, with white curds (H1041) as the control group. Correlation analysis was further conducted between the differential amino acids related to the biosynthesis of chlorophyll (in green curds, H1031), betaxanthin (in yellow curds, H1009), and betacyanin (in gold curds, H1003).

**Fig. 1:**
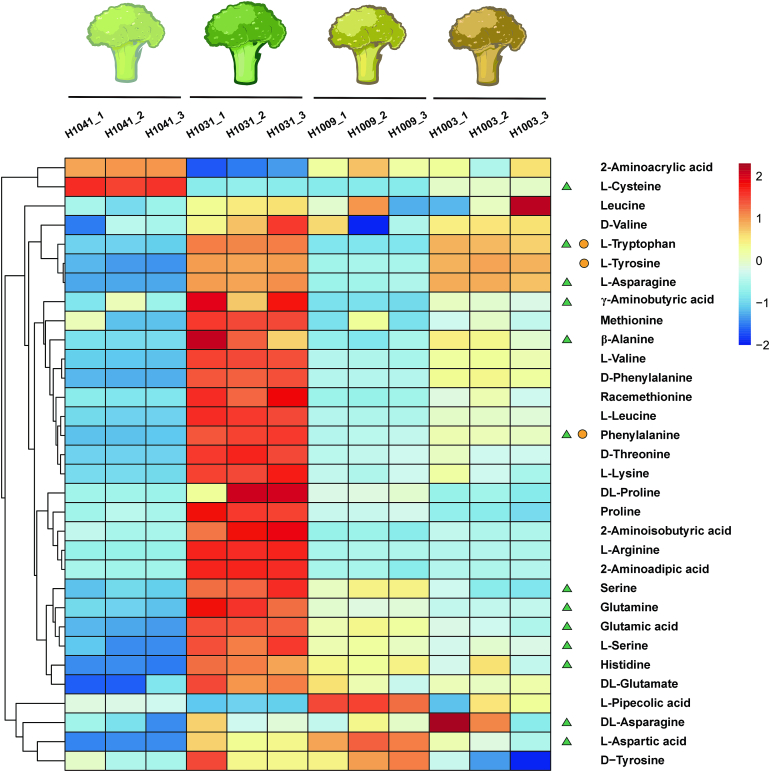
Cluster heatmap of 32 free amino acids in cauliflower curds with different colors. Rows represent 32 free amino acids; columns represent biological replicates of white (H1041), green (H1031), yellow (H1009), and gold (H1003) curds. The color gradient from blue to red indicates the z-score normalized relative abundance (low to high). Green triangles denote chlorophyll-associated amino acids; orange circles denote betalain-associated amino acids. (For interpretation of the references to color in this figure legend, the reader is referred to the web version of this article.)

Cluster heatmap of the 32 free amino acids showed distinct accumulation patterns among four color curds, with intra-group biological replicates clustering tightly (Fig. 1). Green curds (H1031) presented extensive high accumulation of chlorophyll-associated amino acids, such as glutamic acid, glutamine, serine and Histidine (Roca & Pérez-Gálvez, 2021); yellow and gold curds (H1009, H1003) specifically enriched betalain-associated amino acids, including L-tyrosine, phenylalanine and L-tryptophan, which are key precursors for betaxanthin and betacyanin biosynthesis (Miguel, 2018; Strack, Vogt, & Schliemann, 2003); white curds (H1041) exhibited overall low accumulation with only L-cysteine and 2-aminoacrylic acid relatively enriched. The heatmap results further validated the specific correlation between differential amino acids and curd color-associated pigment synthesis.

**Fig. 2:**
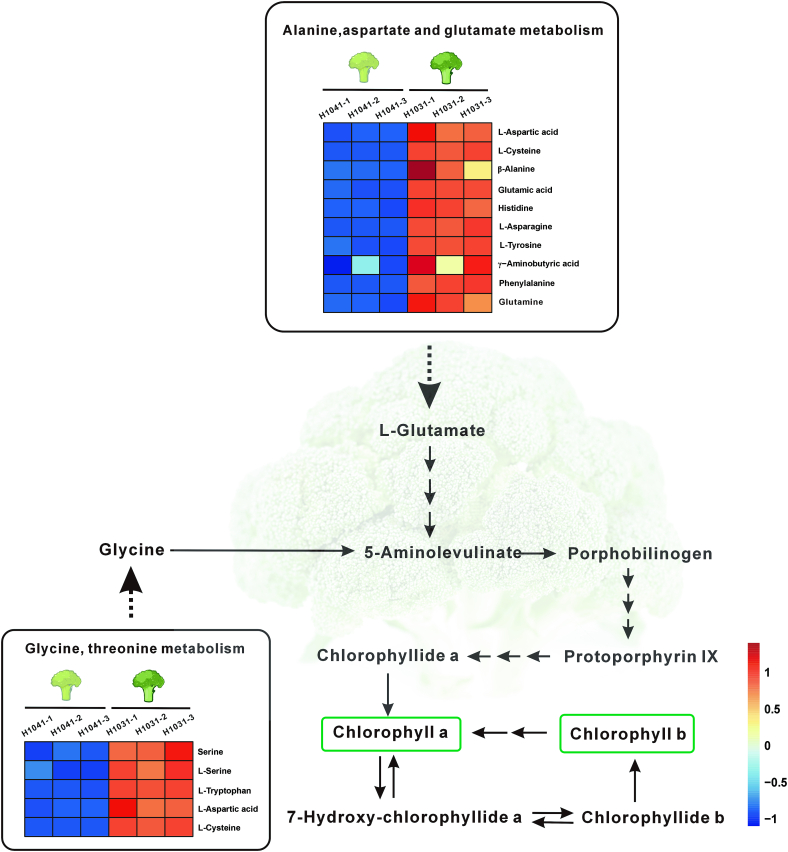
Amino acid metabolism and simplified biosynthetic pathway of chlorophyll in cauliflower curds. The heatmap shows the relative accumulation of key amino acids involved in glycine/threonine (Gly/Thr) and alanine/aspartate/glutamate (Ala/Asp/Glu) metabolism in white (H1041) and green (H1031) curds. Color scale from blue to red indicates low to high relative abundance (z-score normalized). The simplified pathway illustrates the conversion of these amino acids to chlorophyll a and chlorophyll b via the tetrapyrrole biosynthesis pathway. Data are presented as mean of three biological replicates (*n* = 3). Notably, the chlorophyll pigments themselves were not directly detected due to the inherent limitations of untargeted metabolomics; however, the differential accumulation of their upstream amino acid precursors provides reliable evidence for enhanced chlorophyll synthesis capacity in green curds. (For interpretation of the references to color in this figure legend, the reader is referred to the web version of this article.)

For green curds (H1031), compared with the white curd control (H1041), 29 free amino acids were significantly up-regulated, and 3 were down-regulated (Fig. 1). These amino acids showed consistently higher abundance in green curds relative to the white control, suggesting a potential metabolic association with chlorophyll *a*ccumulation in green curds relative to the white control, but not confirming them as the main contributing factors of chlorophyll synthesis. The accumulation patterns of key amino acids involved in glycine/threonine and alanine/aspartate/glutamate metabolism were visualized in Fig. 2. Compared with white curds (H1041), most of these amino acids were significantly up-regulated in green curds (H1031), which are directly linked to chlorophyll *b*iosynthesis. These results suggest that the enhanced amino acid metabolism in green curds provides sufficient precursors (e.g., serine, glutamate, aspartate) to fuel the tetrapyrrole pathway, consistent with the enrichment of chlorophyll-associated precursors in green curds (Beale, 1990; Larkin, 2016; Liu et al., 2022).

**Fig. 3:**
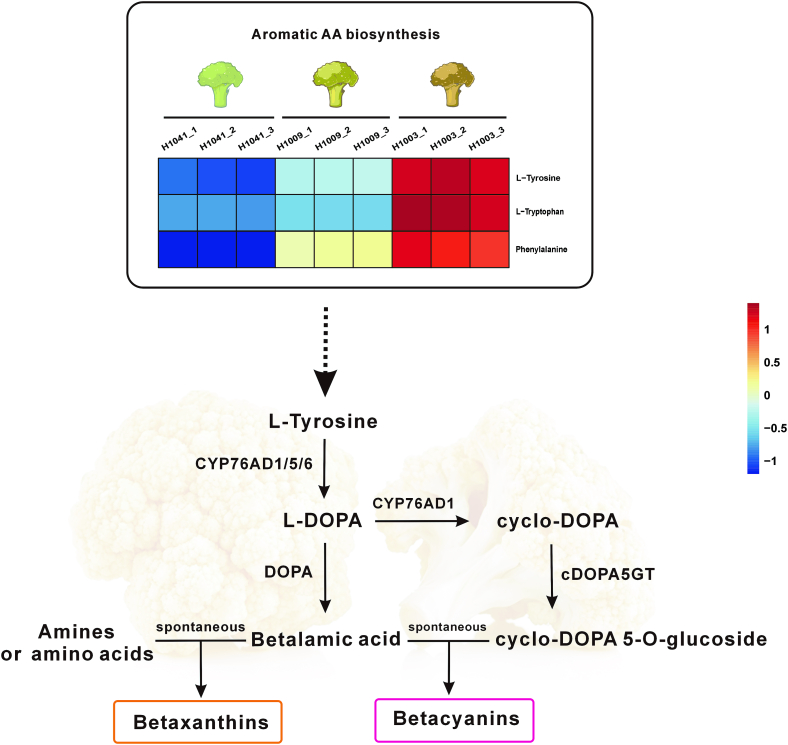
Aromatic amino acid accumulation and its association with betalain precursor metabolism in cauliflower curds. The heatmap shows the accumulation patterns of three aromatic amino acids (tyrosine, phenylalanine, and tryptophan) in white (H1041), yellow (H1009), and gold (H1003) curds. Color scale from blue to red indicates low to high relative abundance (z-score normalized). The simplified pathway illustrates the conversion of L-tyrosine to betaxanthins (yellow pigments) and betacyanins (purple-red pigments) via L-DOPA and betalamic acid. Data are presented as mean of three biological replicates (n = 3). Notably, betalain pigments themselves were not directly detected in this untargeted analysis—a recognized limitation of untargeted LC-MS metabolomics for these compounds; however, the marked upregulation of their precursor amino acids is consistent with enhanced betalain biosynthesis capacity in yellow and gold curds relative to white curds. (For interpretation of the references to color in this figure legend, the reader is referred to the web version of this article.)

Yellow and gold curds (H1009, H1003) exhibited distinct accumulation patterns of aromatic amino acids, which are the primary precursors for betalain biosynthesis, as visualized in Fig. 3. Compared with white curds (H1041), tyrosine, tryptophan, and phenylalanine were significantly upregulated in both yellow and gold curds, with the highest enrichment observed in gold curds. These amino acids are directly linked to the synthesis of betacyanin and betaxanthin, the two major components of betalains (Hanamghar, Russo, Mellor, & Zedler, 2026; X. Zhao, Zhang, Long, Wang, & Yang, 2022). The results suggest that enhanced aromatic amino acid metabolism in yellow and gold curds provides sufficient precursors that may support betalain accumulation, which is associated with the yellow and golden coloration of curds.

To further clarify the metabolic differences underlying distinct pigment accumulation patterns, we compared the amino acid profiles of green curds with yellow and gold curds, as shown in Fig. S6. Yellow and gold curds exhibited a significant upregulation of aromatic amino acids, which are the core precursors for betalain biosynthesis, while green curds showed a specific high accumulation of amino acids involved in glycine/threonine and alanine/aspartate/glutamate metabolism, the key pathways for chlorophyll synthesis. This distinct metabolic partitioning directly explains the differential pigmentation phenotypes: yellow and gold curds prioritize betalain production, whereas green curds channel metabolic flux toward chlorophyll biosynthesis, forming a clear metabolic basis for the color divergence among cauliflower curds (Beale, 1990; Hanamghar et al., 2026; Larkin, 2016; Liu et al., 2022; Roy et al., 2025; X. Zhao et al., 2022).

In summary, using white curds (H1041) as the control, the 32 free amino acids exhibited distinct differential accumulation patterns across green, yellow, and gold cauliflower curds. Specifically, amino acids involved in Gly/Thr and Ala/Asp/Glu metabolism were highly enriched in green curds (H1031), directly fueling chlorophyll biosynthesis (Beale, 1990; Larkin, 2016; Liu et al., 2022); aromatic amino acids (tyrosine, phenylalanine, and tryptophan) were upregulated in yellow (H1009) and gold (H1003) curds, serving as key precursors for betalain (betaxanthin and betacyanin) biosynthesis (Miguel, 2018; H. Yu et al., 2026; X. Zhao et al., 2022). These results highlight distinct amino acid accumulation patterns associated with curd color and provide a metabolic basis for future studies investigating the relationship between amino acid metabolism and nutritional quality in colored cauliflower.

### 3.3 Mechanism of metabolite changes driving nutritional quality improvement

Deeper curd color was associated with coordinated changes in primary and secondary metabolism. The selective enrichment, gradient accumulation, and potential functional synergy of specific metabolites were linked to enhanced antioxidant potential, altered nutritional composition, and distinct functional profiles in colored curds (Alharbi, 2026; Mudondo, Happy, Gang, Ban, & Kang, 2025).

#### 3.3.1 Antioxidant functional potential associated with metabolite accumulation

**Fig. 4:**
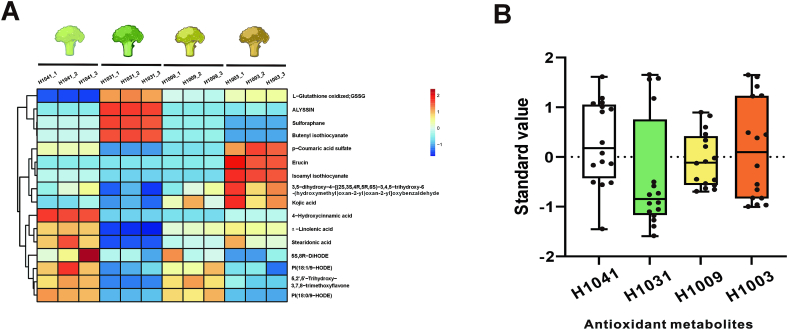
Accumulation patterns of antioxidant metabolites in cauliflower curds with different colors. (A) Heatmap of differentially accumulated antioxidant metabolites, grouped by functional class. (B) Box plot showing the distribution of antioxidant metabolite abundance across color groups (white: H1041, green: H1031, yellow: H1009, gold: H1003; *n* = 3 per group). Individual points represent the standardized value of each detected metabolite. Red = high relative abundance; blue = low relative abundance. (For interpretation of the references to color in this figure legend, the reader is referred to the web version of this article.)

Antioxidant metabolites exhibited distinct color-specific accumulation patterns across white (H1041), green (H1031), yellow (H1009), and gold (H1003) cauliflower curds (Fig. 4). Phenolic acids (e.g., p-coumaric acid sulfate) and flavonoids (e.g., 5,2′,5′-trihydroxy-3,7,8-trimethoxyflavone) exhibited marked color-associated accumulation patterns. Yellow curds (H1009) generally accumulated the highest levels of these compounds, followed by gold (H1003) and green (H1031) curds. Lipid-derived antioxidants (e.g., α-linolenic acid, stearidonic acid, 5S,8R-DiHODE) were distinctly accumulated in white curds, whereas oxidized glutathione (GSSG) was significantly upregulated in green curd. Isothiocyanates, core functional antioxidants in *Brassica* crops (Cedrowski, Grebowski, & Litwinienko, 2022), displayed strict color specificity: sulforaphane, alyssin, and butenyl isothiocyanate were predominantly accumulated in green curds, whereas erucin and isoamyl isothiocyanate peaked in gold curds.

These differential antioxidant metabolites exerted direct free radical-scavenging activity and regulated endogenous antioxidant enzyme expression, forming a dual antioxidant mechanism unique to colored cauliflower (He et al., 2025). The gradient accumulation of phenolic acids and flavonoids in yellow and gold curds, together with the specific enrichment of lipid-derived antioxidants and sulforaphane in green curds, contributed to a robust, color-specific antioxidant network. This network was further strengthened by the synergistic upregulation of lipid-soluble vitamins, making antioxidant potential the most prominent nutritional advantage of colored curds over white curds (Fig. 4).

To further explore the relationships among these metabolites, we performed Pearson correlation analysis based on z-score-normalized relative abundances revealed consistent associations (Fig. S7). The heatmap revealed that metabolites within the same functional classes tended to cluster together, supporting a coordinated, class-level metabolic response to curd color variation. Notably, GABA and glutamic acid showed positive correlations with sulforaphane, consistent with their co-enrichment in green curds. Similarly, aromatic amino acids (tyrosine and phenylalanine) were positively correlated with erucin and p-coumaric acid sulfate, supporting the coordinated accumulation of phenylpropanoid-derived metabolites in yellow and gold curds. These correlation patterns further substantiate the color-associated metabolic shifts described above and suggest that the differential accumulation of bioactive compounds across cultivars is not random but reflects a systematic metabolic shift associated with curd color.

#### 3.3.2 Metabolic changes and regulatory coordination driving nutritional optimization

The global metabolic shift in primary metabolism, coupled with coordinated regulatory signals, is associated with the comprehensive optimization of nutritional composition in colored cauliflower curds.

**Fig. 5:**
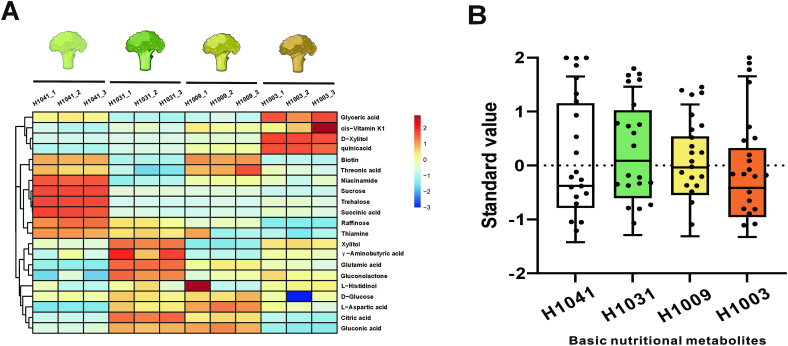
Accumulation patterns of basic nutritional metabolites in cauliflower curds with different colors. (A) Heatmap of differentially accumulated basic nutritional metabolites, grouped by functional class. (B) Box plot showing the distribution of basic nutritional metabolite abundance across color groups (white: H1041, green: H1031, yellow: H1009, gold: H1003; n = 3 per group). Individual points represent the standardized value of each detected metabolite. Red = high relative abundance; blue = low relative abundance. (For interpretation of the references to color in this figure legend, the reader is referred to the web version of this article.)

Compared with white curds (H1041) storing high levels of soluble sugars (sucrose, trehalose and raffinose), colored curds (H1031, H1009, H1003) exhibited a significant decline in sugar storage, accompanied by the upregulation of TCA cycle intermediates (e.g., citric acid, succinic acid) and other organic acids (e.g., gluconic acid). This shift is consistent with increased carbon flux toward respiratory metabolism and secondary metabolite synthesis, which may support the energy demand associated with pigment accumulation in colored curds (Landi, Zivcak, Sytar, Brestic, & Allakhverdiev, 2020; Selwal et al., 2023). In nitrogen metabolism, functional amino acids (e.g., GABA, glutamic acid) were specifically enriched in colored genotypes, while biotin and niacinamide showed distinct distribution patterns across color groups, reflecting the nutritional diversity associated with curd pigmentation (Fig. 5).

**Fig. 6:**
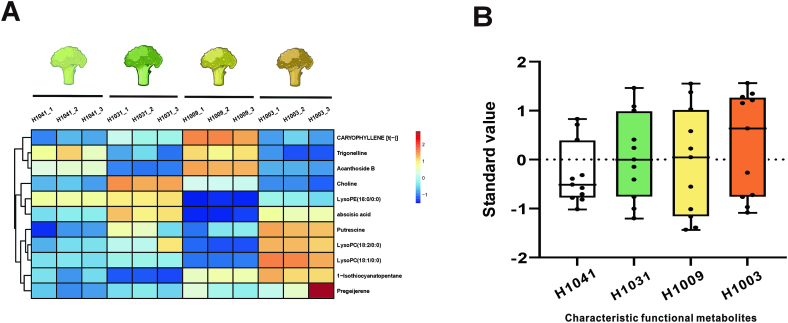
Accumulation patterns of characteristic functional metabolites in cauliflower curds with different colors. (A) Heatmap of differentially accumulated characteristic functional metabolites, grouped by functional class. (B) Box plot showing the distribution of basic nutritional metabolite abundance across color groups (white: H1041, green: H1031, yellow: H1009, gold: H1003; n = 3 per group). Individual points represent the standardized value of each detected metabolite. Red = high relative abundance; blue = low relative abundance. (For interpretation of the references to color in this figure legend, the reader is referred to the web version of this article.)

Lipid-related metabolites and signaling molecules formed a synergistic network to support nutritional quality. Lysophospholipids (LysoPC, LysoPE) were extensively remodeled in colored curds, particularly in gold curds (H1003) (Fig. 6), providing essential membrane components and facilitating the transport and accumulation of fat-soluble vitamins (e.g., cis-vitamin K1) (Fig. 5). Furthermore, regulatory metabolites such as abscisic acid (ABA) and putrescine displayed color-specific enrichment (Fig. 6): ABA in green curds may contribute to chloroplast stability and nutrient synthesis (Wei, Javed, Liu, Ali, & Gao, 2025), while putrescine in gold curds optimized nitrogen utilization and accelerated the production of nitrogen-containing functional nutrients (Sasikumar & Nampoothiri, 2025).

Collectively, the coordinated changes of primary metabolism and regulatory signaling networks transformed colored curds from simple sugar storage organs into balanced, nutrient-enriched tissues, reflecting the superior nutritional foundation observed in colored cauliflower varieties.

#### 3.3.3 Unique functional nutritional advantages by characteristic functional metabolite diversification

Characteristic functional metabolites, including flavor compounds, signaling molecules, and bioactive secondary metabolites, exhibited distinct color-specific accumulation patterns, endowing different colored curds with unique functional advantages (Fig. 6). Isothiocyanates were the main contributors to functional diversity (Cedrowski et al., 2022): high sulforaphane accumulation in green curds enhanced anti-inflammatory, antioxidant, and detoxification activities (Treasure, Harris, & Williamson, 2023), while specific erucin enrichment in gold curds significantly improved anticancer and anti-oxidative stress capacities (Bello et al., 2023) (Fig. 4).Terpenoid metabolites (e.g., caryophyllene, pregeijerene) showed color-specific enrichment, with the highest levels in yellow and gold curds, adding anti-inflammatory and antibacterial attributes while enriching curd flavor (Patil, Baviskar, Dinore, & Yelwande, 2025) (Fig. 6). They synergized with isothiocyanates to shape the unique flavor profile of colored curds.

Other functional secondary metabolites were differentially distributed across genotypes (Fig. 6): trigonelline and acanthoside B were predominantly accumulated in yellow curds, conferring hypoglycemic and immunomodulatory potential, respectively. Signaling molecules such as ABA and putrescine displayed distinct color-specific enrichment, further modulating stress tolerance and nutrient accumulation (Sasikumar & Nampoothiri, 2025; Wei et al., 2025).

The diversification of characteristic functional metabolites contributed to a “flavor-function integration” profile in colored curds, which was associated with the metabolic changes linked to nutritional differentiation in cauliflower. The key nutritional biomarkers associated with each curd color are summarized in Table 1.

**Table 1:** Key nutritional biomarkers associated with each curd color in cauliflower.

Curd color	Key metabolite classes	Representative biomarkers	Main functional implications
White (H1041)	Soluble sugars; Organic acids; Vitamins; Phenolic compounds (flavonoids and phenolic acids);	Sucrose, Trehalose, Raffinose; Succinic acid; Biotin, Niacinamide; p-Coumaric acid sulfate, 5,2′,5′-Trihydroxy-3,7,8-trimethoxyflavone	Energy-storage mode; basal energy supply; vitamin-based and flavonoid-based antioxidant potential
Green (H1031)	Chlorophyll-associated amino acids; Isothiocyanates; Neuroactive metabolites; Sugar alcohols	Glutamic acid, Glutamine, Serine; Sulforaphane, Alyssin, Butenyl isothiocyanate; GABA; Xylitol	Chlorophyll precursors; anti-inflammatory/antioxidant; neuroprotective potential
Yellow (H1009)	Flavonoids; Lipid-derived antioxidants; Vitamins	5,2′,5′-Trihydroxy-3,7,8-trimethoxyflavone; 5S,8R-DiHODE, PI(18:1/9-HODE), PI(18:0/9-HODE); Biotin	Dietary flavonoids; lipid-derived antioxidant potential; vitamin-based antioxidant potential
Gold (H1003)	Phenolic compounds (flavonoids and phenolic acids); Isothiocyanates; Flavor-related metabolites	p-Coumaric acid sulfate; Erucin, Isoamyl isothiocyanate; Pregeijerene	Broad-spectrum antioxidant; anticancer potential; flavor diversity

Representative metabolites were selected based on their differential accumulation patterns and functional relevance as discussed in the main text. GABA, γ-aminobutyric acid.

## 4 Discussion

**Fig. 7:**
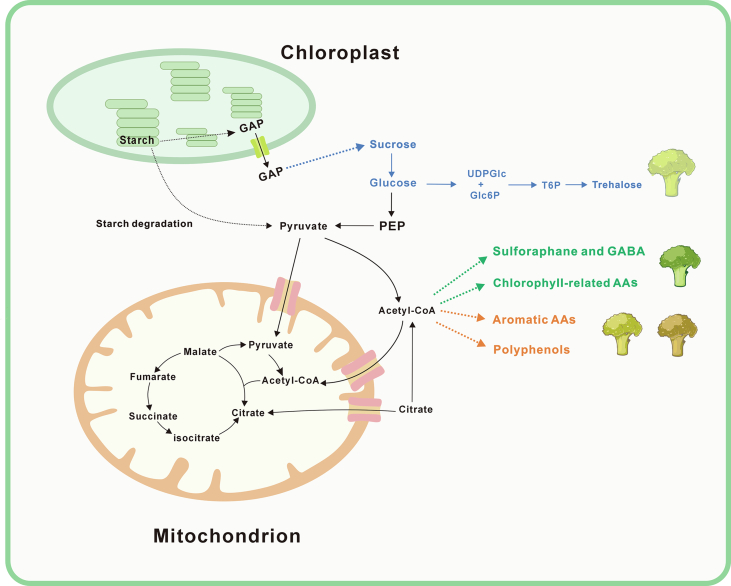
Schematic illustration of primary carbon metabolism and its linkage to color-associated secondary metabolite and antioxidant potential differences in cauliflower curds. The diagram compares white (H1041) and colored (green H1031, yellow H1009 and gold H1003) curds, highlighting shifts from sugar accumulation (white) toward increased TCA cycle and secondary metabolite biosynthesis (colored). Starch degradation in chloroplasts produces glyceraldehyde-3-phosphate (GAP), which is partitioned into sucrose and trehalose synthesis (via glucose-6-phosphate (Glc6P), uridine diphosphate glucose (UDPGlc), and trehalose-6-phosphate (T6P)) or glycolysis to pyruvate via intermediates including phosphoenolpyruvate (PEP). Pyruvate enters the mitochondria for the TCA cycle, generating acetyl-coenzyme A (acetyl-CoA). Acetyl-CoA feeds into the biosynthesis of chlorophyll-related amino acids, sulforaphane, GABA, aromatic amino acids, and polyphenols in different cauliflower varieties, which is consistent with enhanced antioxidant potential. (For interpretation of the references to color in this figure legend, the reader is referred to the web version of this article.)

In this study, non-targeted metabolomics was used to systematically characterize metabolic profiles in four cauliflower cultivars with distinct curd colors (white, green, yellow, and gold). A total of 2685 metabolites were identified, and multivariate analysis revealed significant metabolic divergence among color groups, indicating extensive metabolic remodeling associated with curd pigmentation. These findings provide a global metabolic framework for understanding color-associated differentiation in nutritional quality and metabolite networks. A proposed working model summarizing the overall metabolic changes is illustrated in Fig. 7.

### 4.1 Amino acid metabolism reflects phenotypic divergence but is not the primary driver of color formation

Amino acids serve as important precursors for pigment biosynthesis(Park et al., 2013; Roy et al., 2025; Sheng et al., 2025): green curds showed higher levels of amino acids linked to chlorophyll synthesis (Fig. 2), while yellow and gold curds accumulated more aromatic amino acids (e.g., L-Tyrosine, Phenylalanine) that are precursors for betalain (Fig. 3).

It is important to acknowledge that the absence of direct pigment detection is a known technical constraint of non-targeted LC-MS metabolomics, which typically has limited sensitivity for low-abundance, poorly ionizing, or unstable pigment molecules. This limitation does not invalidate our findings, as the upstream amino acid precursors we identified are well-established in the literature as the committed substrates for chlorophyll and betalain biosynthesis (Beale, 1990; Tanaka et al., 2008).

Although we did not directly quantify pigments, the interpretation of precursor accumulation as an indicator of biosynthetic capacity is supported by the established relationship between precursor availability and pathway flux in plant specialized metabolism. In the tetrapyrrole and betalain pathways, the supply of committed substrates—glutamate for chlorophyll and tyrosine for betalains—is a recognized determinant of downstream pigment production (Hanamghar et al., 2026; Peng et al., 2025; X. Zhao et al., 2022). Thus, the observed enrichment of these precursors in specific color groups is consistent with, and provides a metabolomic basis for, differential pigment synthesis capacity. Notably, although betalains are more commonly reported in Caryophyllales, the core biosynthetic pathway genes have also been identified in Brassicaceae species, as annotated in the KEGG database (e.g., the betalain biosynthetic pathway in *Brassica rapa*; brp00965) (Kanehisa, Furumichi, Sato, Kawashima, & Ishiguro-Watanabe, 2023), supporting the relevance of discussing betalain precursor metabolism in cauliflower. Our inference is limited to the precursor level and does not imply detection of the downstream pigments themselves.

Our correlation analyses between these precursor amino acids and visual color phenotypes provide a metabolic link that is consistent with the current understanding of plant pigment biosynthesis (Huang et al., 2021). Therefore, while causality cannot be established from our data, the observed precursor enrichment patterns serve as a reliable indicator of differential pigment synthesis capacity among color groups. Thus, while these amino acid differences are metabolic correlates rather than the primary cause of color formation, they may indirectly influence downstream pigment synthesis efficiency. However, causal relationships remain unproven and require further validation to confirm whether manipulating amino acid levels directly alters curd color.

### 4.2 Primary metabolic changes support secondary metabolism and nutritional quality

Beyond amino acid metabolism, widespread changes of primary metabolism was observed in colored curds. Compared with white curds, which accumulated high levels of soluble sugars, colored cauliflowers showed reduced sugar storage, enhanced TCA cycle activity and organic acid metabolism (Fig. 5). This shift indicates increased carbon flux toward energy production and secondary metabolite synthesis, which is consistent with the high metabolic demand of pigment accumulation and antioxidant pathway activation (Afzal, Chaudhary, & Singh, 2021; T. Zhao et al., 2025).

Accumulation patterns of lipid-derived antioxidants and fat-soluble vitamins also differed among color groups, contributing to the distinct nutritional characteristics of colored curds.

### 4.3 Secondary metabolic divergence underlies flavor and functional nutritional characteristics

Secondary metabolites, particularly flavonoids, phenolic acids, terpenoids, and isothiocyanates, exhibited strong color-specific accumulation patterns. Phenolic acids and flavonoids showed distinct color-associated accumulation patterns, with the highest levels of phenolic acids in gold curds and flavonoids in yellow curds. (Hernández-Rodríguez, Baquero, & Larrota, 2019; Mutha, Tatiya, & Surana, 2021). Isothiocyanates showed strict color specificity: sulforaphane was highly enriched in green curds, while erucin dominated in gold curds (Fig. 4). These compounds are closely associated with antioxidant, anti-inflammatory, and potential anticancer activities, contributing to the superior functional quality of colored cauliflower (Bello et al., 2023; Cedrowski et al., 2022; Treasure et al., 2023).

Sesquiterpenoid derivatives, such as pregeijerene, were detected in yellow and gold curds and acted synergistically with isothiocyanates to shape unique flavor profiles (Fig. 4; Fig. 6). Together, these secondary metabolic differences distinguish color phenotypes and are closely associated with the nutritional and functional diversity of cauliflower curds (Patil et al., 2025; Treasure et al., 2023).

### 4.4 Regulatory metabolites coordinate metabolic homeostasis during color formation

Several regulatory metabolites, including ABA, putrescine, choline derivatives, and lipid signaling molecules, were differentially accumulated in a color-specific manner (Fig. 6). ABA was enriched in green curds and may contribute to chloroplast stability and stress tolerance. Putrescine was upregulated in gold curds and may enhance nitrogen utilization and secondary metabolism. Lipid signals and methyl metabolism intermediates help maintain metabolic balance and improve the stability of functional metabolites. These regulatory components form a coordinated network that supports sustained nutrient accumulation and quality improvement (Sana, Aftab, Naeem, Jha, & Prasad, 2025; Sasikumar & Nampoothiri, 2025; Wei et al., 2025).

### 4.5 Implications for cauliflower as a functional food

The following discussion focuses on the potential dietary relevance of the identified metabolites and does not imply therapeutic applications. The color-associated metabolic differences identified in this study have direct implications for the use of cauliflower as a functional food ingredient. Yellow and gold curds (H1009, H1003), with their elevated levels of flavonoids and phenolic acids, may serve as natural sources of dietary antioxidants (Hernández-Rodríguez et al., 2019; Mutha et al., 2021). Green curds (H1031), characterized by high sulforaphane accumulation, could be targeted for the development of anti-inflammatory functional foods (Park et al., 2013; Sheng et al., 2025; Treasure et al., 2023; H. Yu et al., 2026). Beyond its well-recognized anti-inflammatory properties, sulforaphane has attracted increasing interest for its potential neuroprotective effects, largely attributed to its capacity to modulate oxidative stress and inflammatory signaling cascades (Wu et al., 2025). Recent studies have highlighted that selenium-based interventions can suppress oxidative damage and inflammatory pathways, including mTOR- and JAK/STAT-associated mechanisms, in the context of cerebrovascular and neurodegenerative disorders (Ding et al., 2026). While our study did not investigate neurological outcomes, the marked accumulation of sulforaphane in green curds points to their potential dietary relevance for mitigating oxidative and inflammatory processes. This observation further supports the valorization of green cauliflower as a functional ingredient in neuroprotective nutritional strategies. The distinct isothiocyanate profiles across color groups also suggest that color can be used as a visual indicator for specific bioactive compound profiles in cauliflower breeding and marketing. These findings support the notion that color variation in cauliflower is not merely aesthetic but carries meaningful nutritional implications, enabling evidence-based recommendations for consumers seeking specific health benefits.

From a breeding perspective, the color-specific metabolic signatures identified in this study provide a direct rationale for selecting parental lines based on curd color to enhance specific bioactive compound profiles. For example, breeding programs targeting sulforaphane-rich varieties could prioritize green germplasm, while those aiming for high flavonoid or phenolic acid content could focus on yellow and gold accessions. This color-based selection strategy offers a simple and cost-effective visual marker for nutritional quality improvement, which can be readily integrated into existing cauliflower breeding pipelines (René, Noel, Damien, & Sidiky, 2024).

For functional food development, the distinct metabolic profiles across color groups suggest that different cauliflower varieties can be positioned for specific health-oriented markets. Green curds, with their high sulforaphane and GABA content, may be suitable for products targeting anti-inflammatory or neuroprotective applications. Yellow and gold curds, rich in flavonoids and phenolic acids, could be developed as natural antioxidant ingredients for dietary supplements or functional beverages (Phupaboon et al., 2025). The integration of metabolomics-based quality assessment with color-guided selection thus offers a practical framework for both breeders and food processors to develop cauliflower varieties with optimized nutritional value and targeted health benefits.

While the bioactive compounds identified in colored cauliflower curds—particularly isothiocyanates such as sulforaphane—are generally recognized for their health-promoting effects, it is also important to consider potential safety aspects associated with their consumption. Isothiocyanates are bioactive and, at high doses, may exhibit genotoxic potential or interfere with thyroid function through their goitrogenic effects, as has been documented for glucosinolate derivatives in cruciferous vegetables (Kumar, 2020). Toxicological evaluation of isothiocyanates in food systems has highlighted the importance of understanding both their beneficial bioactivities and potential risks at elevated exposure levels (Nadeem & Mahmood, 2025). Furthermore, plant-based foods inherently contain a range of nutrients and anti-nutritional factors, and understanding the balance between beneficial compounds and potentially limiting constituents is essential for comprehensive safety assessment (Abidinsyah & Yaakub, 2025). However, these effects are primarily associated with excessive intake or extreme dietary imbalances, and the levels typically consumed through a balanced diet are considered safe. The present study does not investigate toxicological thresholds, and further research is needed to establish safe and effective dosage ranges for the development of color-specific functional food products.

### 4.6 Future perspectives

The non-targeted metabolomics approach employed in this study demonstrates considerable potential for industrial application in color-specific variety selection. The color-associated metabolic signatures identified here can serve as a rapid screening tool for breeding programs, enabling the early selection of varieties with desired bioactive compound profiles based on curd color. This approach reduces the reliance on time-consuming and costly targeted chemical analyses during the initial stages of breeding pipelines, thereby accelerating the development of nutritionally enhanced cauliflower varieties. However, several inherent limitations of non-targeted metabolomics should be acknowledged. Data processing and interpretation in untargeted LC-MS metabolomics remain challenging due to the complexity and high dimensionality of the data (Gorrochategui et al., 2016). First, the semi-quantitative nature of the data does not provide absolute concentrations of metabolites, which may limit direct comparability across different studies or batches. Second, low-abundance pigments such as chlorophylls and betalains are often poorly detected due to ionization efficiency issues, as noted in this study. Third, the metabolomic profile is influenced by environmental factors (e.g., growing conditions, harvest time), and the biomarkers identified in this study require validation across multiple seasons and locations before they can be reliably applied in industrial breeding programs. Despite these limitations, non-targeted metabolomics remains a powerful hypothesis-generating tool that, when combined with targeted validation, can effectively support color-guided nutritional breeding (Souza & Patti, 2021). Furthermore, postharvest processing of colored cauliflower varieties should be optimized to preserve their unique bioactive profiles, as processing conditions such as fermentation and drying can significantly affect the physical, chemical, and sensory characteristics of food products (Flores et al., 2025). The nutraceutical potential of plant-derived extracts further supports the use of color-specific cauliflower varieties as ingredients in functional food formulations targeting specific health outcomes (Wasim Sajid, Nadeem, Akhtar, & Din, 2025).

While this study offers a comprehensive metabolic overview of color-related differentiation in cauliflower, several key areas require further research: targeted quantitative analysis of low-abundance pigments (chlorophylls, betaxanthins, betacyanins) is a key priority for future research to directly connect metabolite profiles with color phenotype (Park et al., 2013; Roy et al., 2025; Sheng et al., 2025; Shubham Singh et al., 2021; Tanaka et al., 2008); the causal link between amino acid precursors and pigment synthesis remains unclear and should be explored using stable isotope tracing, gene editing, and enzyme activity assays; integrated transcriptomic–metabolomic analysis is necessary to identify key genes and regulatory factors controlling color formation and nutritional quality; postharvest stability and processing effects on functional metabolites need evaluation for practical application; and in vitro and in vivo experiments are required to validate the bioavailability and health-promoting effects of color-specific metabolites to facilitate the development of high-value functional cauliflower products.

### 4.7 Conclusions

In conclusion, curd color differentiation in cauliflower is accompanied by extensive metabolic changes involving primary and secondary metabolism, regulatory signaling, and nutrient accumulation. Amino acid precursors associated with chlorophyll and betalain synthesis were differentially accumulated, but these changes represent metabolic correlates rather than primary drivers of color formation, and key pigments were not directly detected due to methodological limitations. The observed metabolic shifts were associated with enhanced antioxidant potential, flavor diversity, and nutritional quality in colored curds. Although the sample size (*n* = 3 per cultivar) is consistent with standard practice in non-targeted metabolomics, we acknowledge that broader validation across seasons, locations, and growing conditions would further strengthen the confidence in these biomarkers. Future studies with larger sample sets and multi-environment trials will be valuable to assess the stability of the color-associated metabolic signatures reported here. This study provides a metabolic foundation for understanding color-related quality differentiation in cauliflower and supports the evidence-based utilization of color-specific varieties in functional food development and nutritional breeding programs.

## CRediT authorship contribution statement

**Difei Wang:** Writing – original draft, Visualization, Validation, Methodology, Formal analysis, Data curation. **Meijiao Lu:** Validation, Methodology. **Zitong Zhao:** Validation, Methodology. **Laibin Cheng:** Validation, Methodology. **Wanqing Dong:** Validation, Methodology. **Jin Cui:** Resources, Methodology. **Zhenguo Shen:** Writing – review & editing, Supervision, Project administration. **Nana Su:** Writing – review & editing, Supervision, Resources, Project administration, Methodology, Funding acquisition, Conceptualization.

## Declaration of competing interest

The authors declare that they have no known competing financial interests or personal relationships that could have appeared to influence the work reported in this paper.

## Data Availability

The raw LC-MS data and processed metabolite tables are available from the corresponding author upon reasonable request.
