# Supplementary material for: Color-associated metabolic divergence and nutritional variation in colored cauliflower curds
Source: Food Chem X. 2026 Jul 9;37:104197. doi: 10.1016/j.fochx.2026.104197 (PMC13382464; doi:10.1016/j.fochx.2026.104197)
Supplement: Supplementary file 1 — Supplementary material [file mmc1.docx]

**Supplementary Information**

**
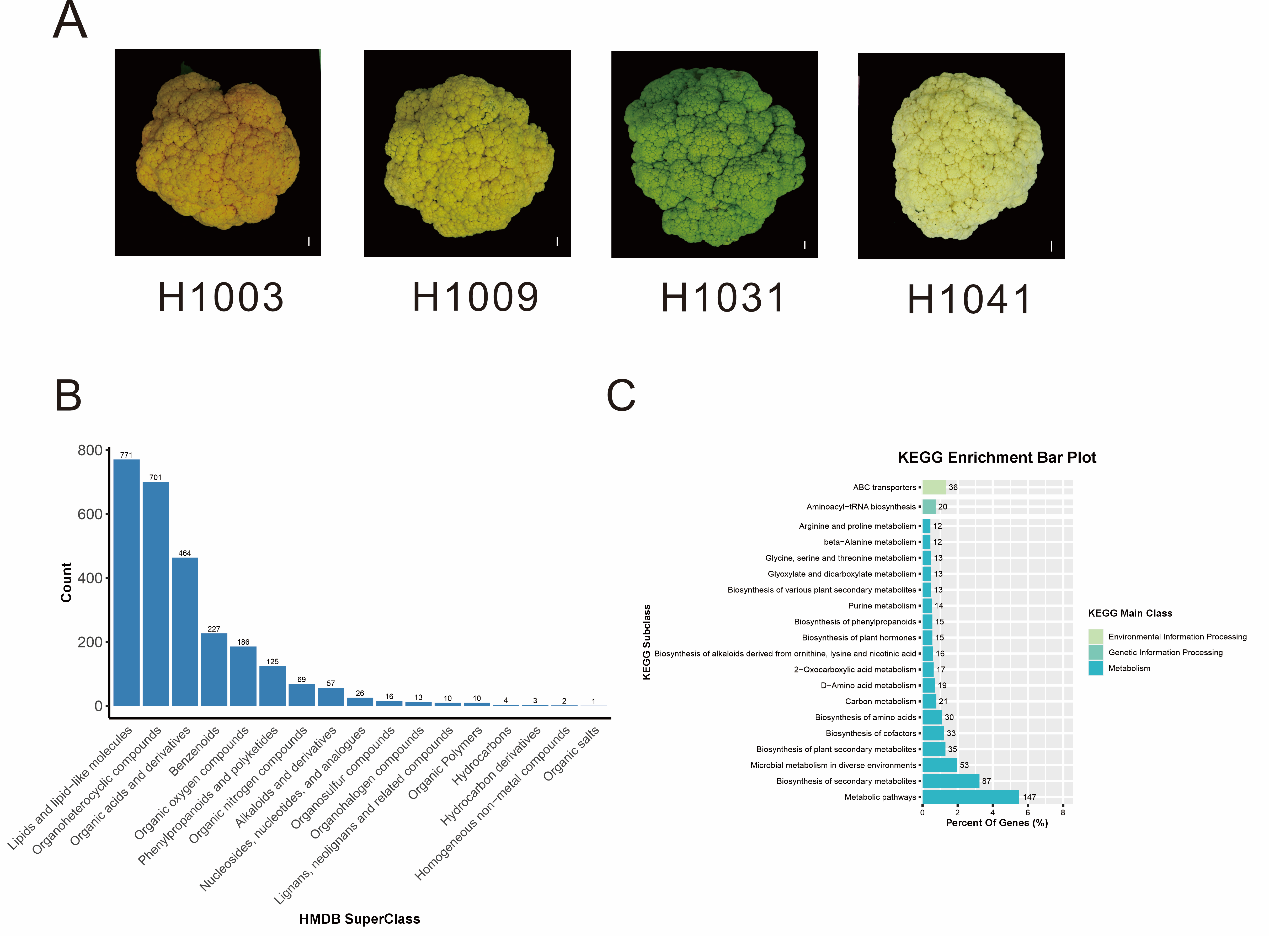
**

**Figure S1. Metabolomic characterization of cauliflower curds with different colors.** (A) Phenotypes of four cauliflower curds with distinct colors. Scale bar = 1 cm. (B) HMDB superclass classification and annotation statistics of all identified metabolites. (C) Top 20 KEGG enrichment pathways of metabolites in different color curd samples.


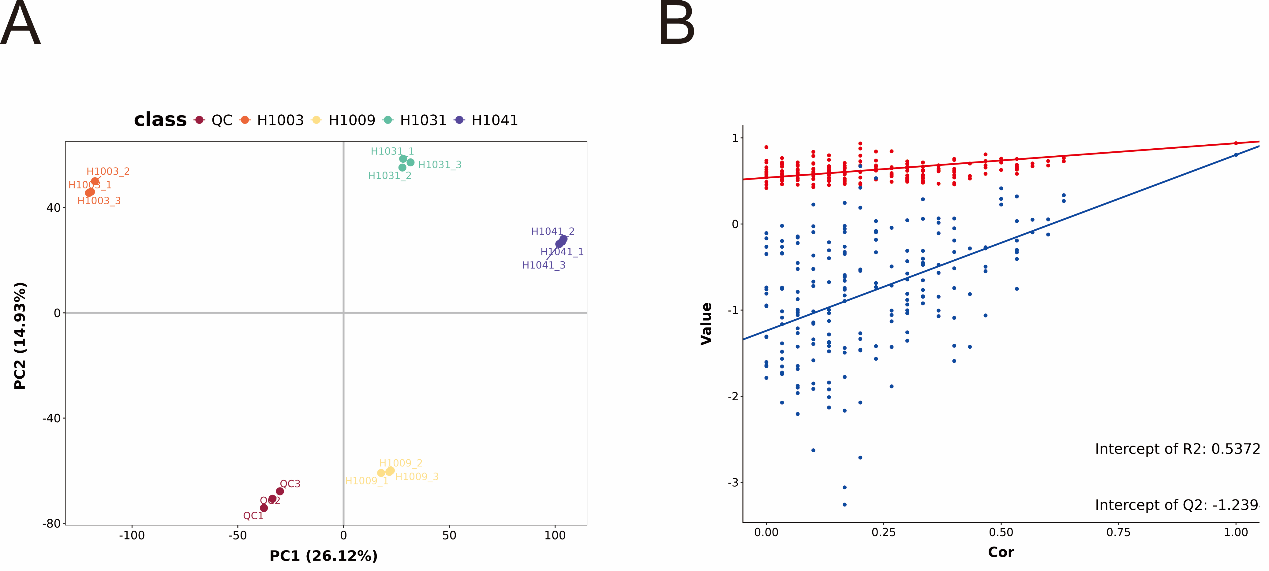


**Figure S2. Metabolomic profiling and PLS-DA model validation of cauliflower curds.** (A) PLS-DA score plot. PC1 and PC2 explain 26.12% and 14.93% of the total metabolic variation, respectively. Colors: red = QC; orange = gold (H1003); yellow = yellow (H1009); green = green (H1031); purple = white (H1041). (B) Permutation test of the PLS-DA model (200 permutations). R² and Q² intercepts are 0.5372 and −1.2394, confirming no overfitting.


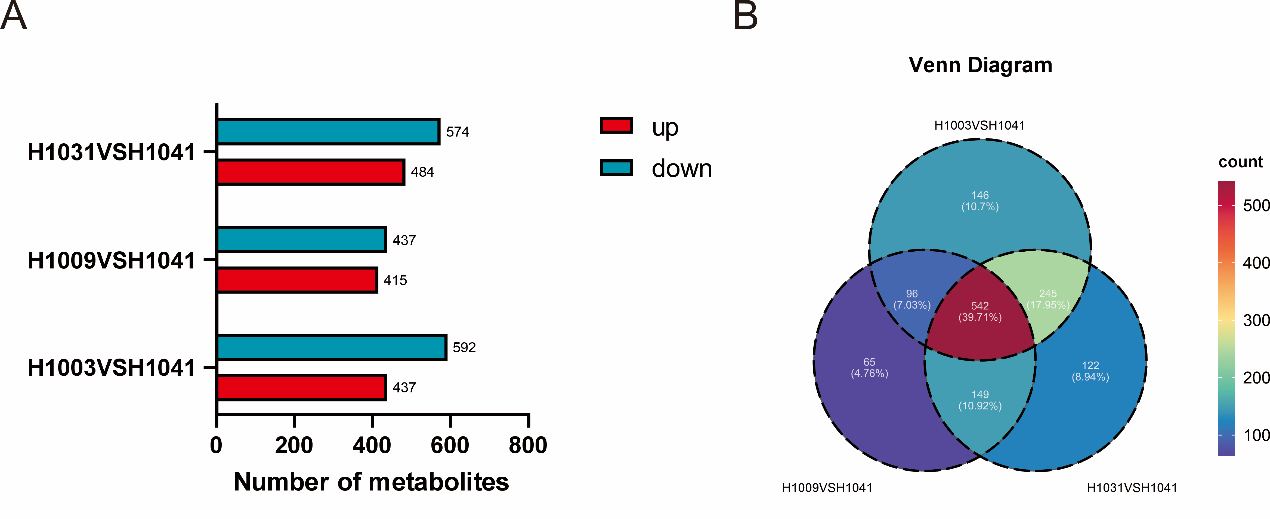


**Figure S3. Differential metabolite analysis of colored curds relative to white control (H1041).** (A) Number of significantly up-regulated (red) and down-regulated (blue) metabolites in gold (H1003), yellow (H1009), and green (H1031) curds. (B) Venn diagram showing the overlap of differentially accumulated metabolites (DAMs) among three colored groups.


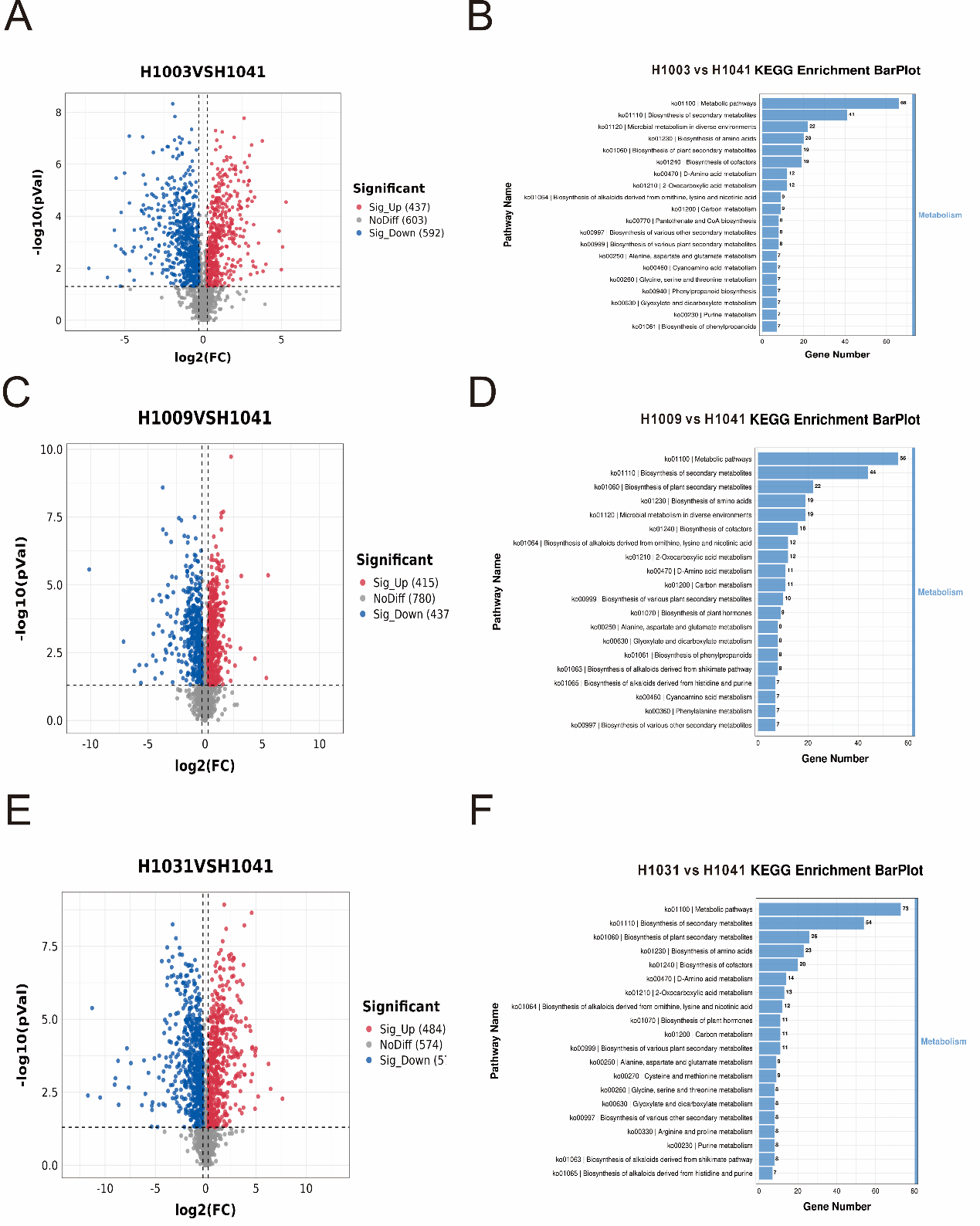


**Figure S4. Differential analysis and KEGG enrichment in colored curds vs. white control.** (A, C, E) Volcano plots of DAMs in green (H1031), yellow (H1009), and gold (H1003) curds. Red = up-regulated; blue = down-regulated; gray = no significant difference. (B, D, F) KEGG enrichment bubble plots of DAMs in the three groups. Bubble size = DAM number; color = significance (*p*-value).


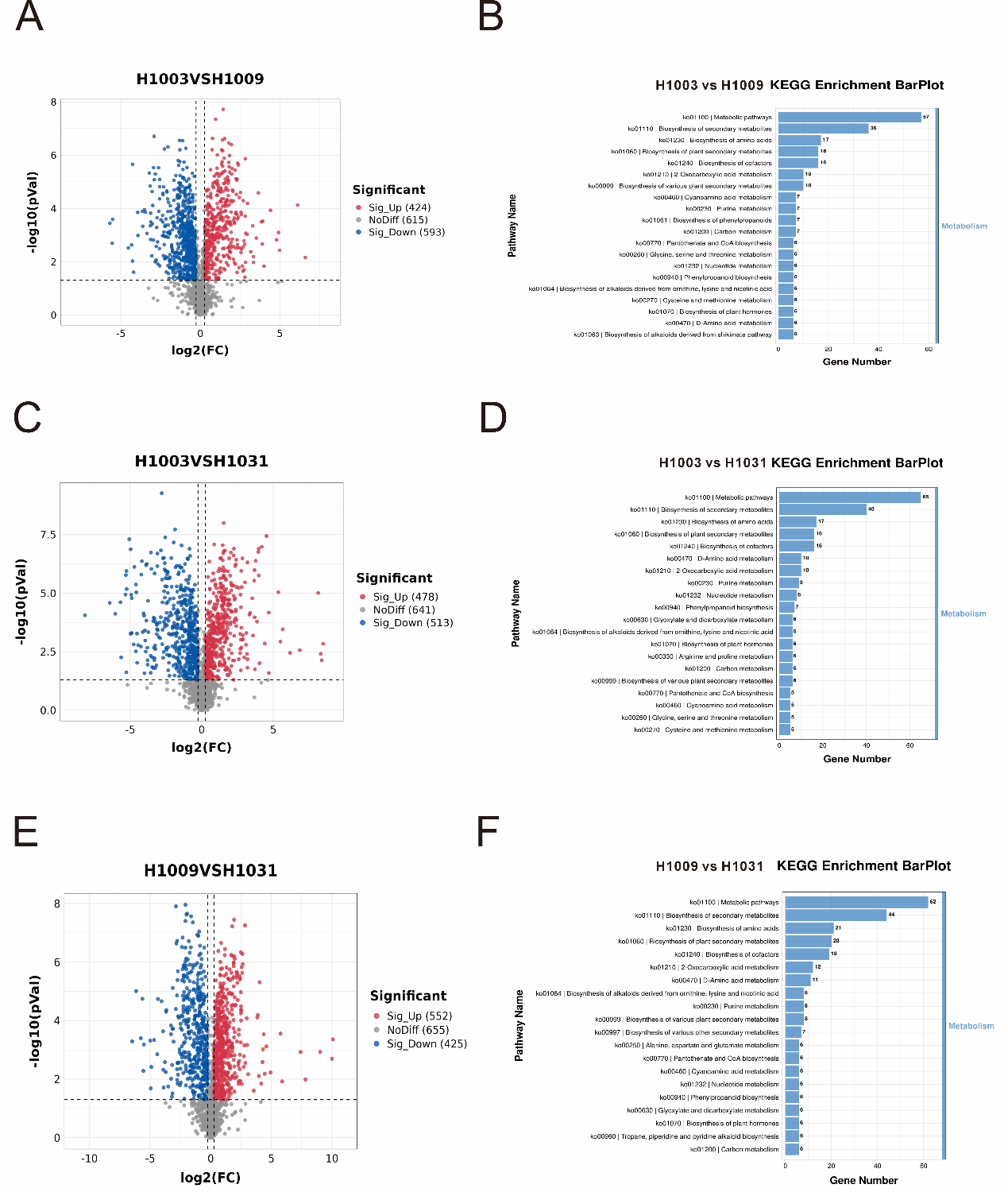


**Figure S5. Differential metabolite analysis and KEGG enrichment among three colored curds.** (A, C, E) Volcano plots of DAMs in H1003 vs H1009, H1003 vs H1031, and H1009 vs H1031. Red = up-regulated; blue = down-regulated; gray = no significant difference. (B, D, F) KEGG enrichment bubble plots of DAMs in the three pairwise comparisons. Bubble size = DAM number; color = significance (*p*-value).


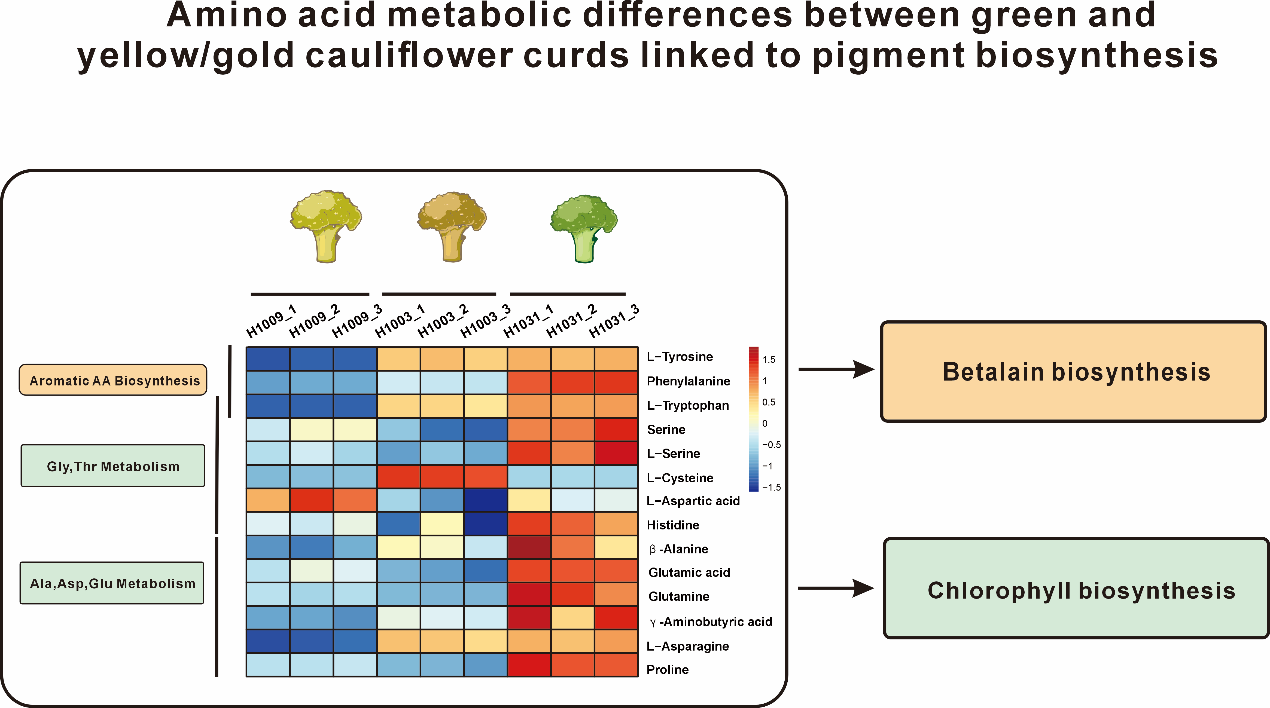


**Figure S6. Amino acid metabolic differences between green and yellow/gold cauliflower curds.** Heatmap shows z-score normalized amino acid profiles in yellow (H1009), gold (H1003), and green (H1031) curds (blue = low, red = high). Aromatic amino acids are enriched in yellow/gold curds for betalain biosynthesis; amino acids in Gly/Thr and Ala/Asp/Glu metabolism are enriched in green curds for chlorophyll biosynthesis.

**
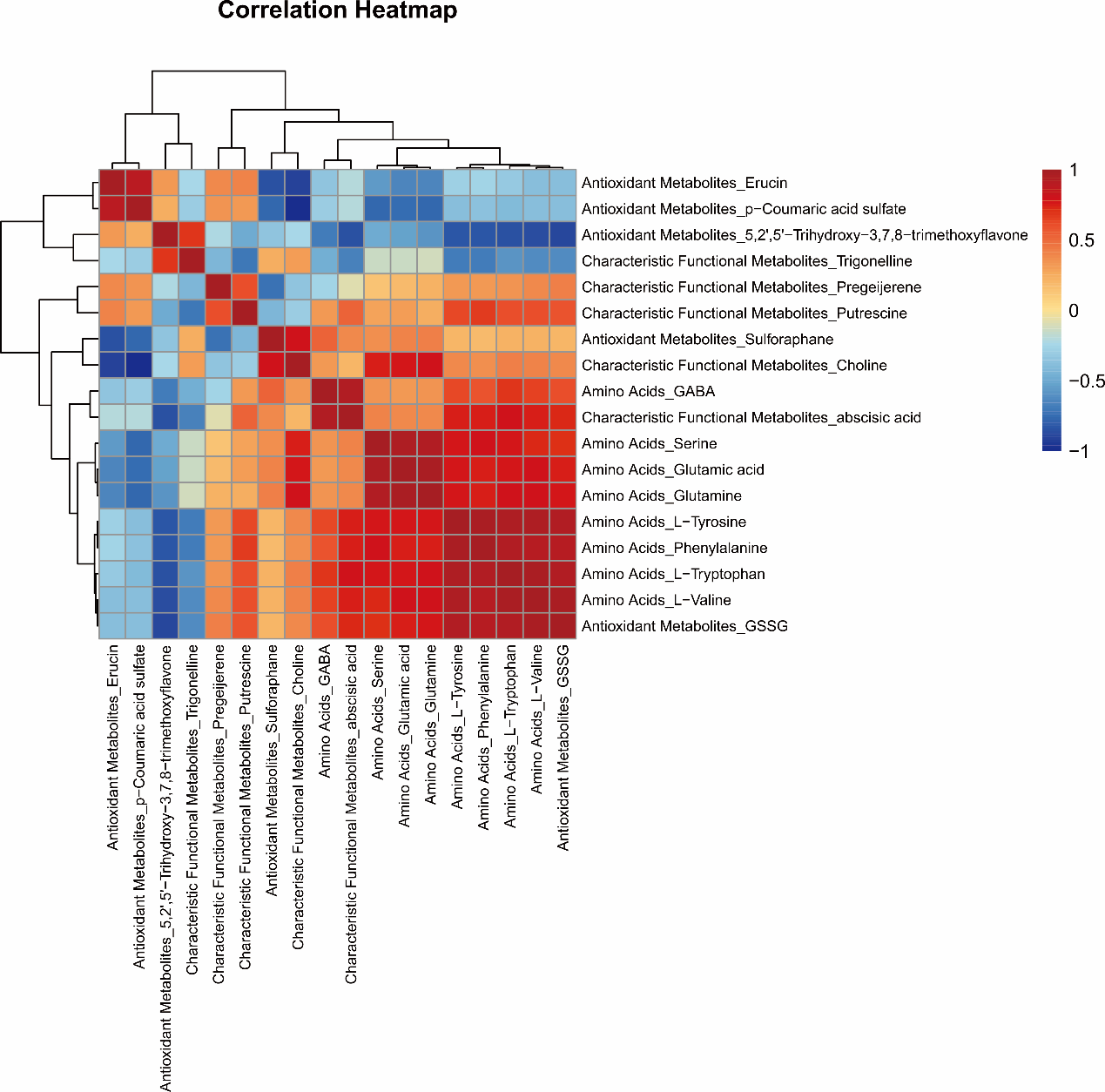
**

**Figure S7. Correlation heatmap between key amino acids and antioxidant/functional metabolites in cauliflower curds.** Pearson correlation coefficients were calculated among 17 selected metabolites (eight amino acids, five antioxidant metabolites, and four functional metabolites) based on their Z‑score‑normalized relative abundances across four cultivars (white H1041, green H1031, yellow H1009, gold H1003). Red indicates positive correlation; blue indicates negative correlation. The color intensity represents the correlation strength from −1.0 to 1.0. Metabolite labels are listed in the legend.
